# Tea polysaccharides improved the water mobility, thermal stability and microstructural changes of *Hypophthalmichthys molitrix* surimi during frozen thaw cycles

**DOI:** 10.1016/j.fochx.2026.104498

**Published:** 2026-09-22

**Authors:** Noman Walayat, Piotr Kulawik, Isam A. Mohamed Ahmed, Asad Nawaz, Nadia Niaz, Mahmoud Salah, Muhammad Naveed Sheas, Zhucheng Su, Ran Wei

**Affiliations:** a College of Tea Science and Tea Culture, Zhejiang Agriculture and Forestry University, 311400 Hangzhou, PR China; b Department of Animal Products Processing, University of Agriculture, Balicka 122, 30-149 Kraków, Poland; c Department of Food Science and Nutrition, College of Food and Agricultural Sciences, King Saud University, P.O. Box 2460, Riyadh 11451, Saudi Arabia; d College of Chemistry and Bioengineering, Hunan University of Science and Engineering, Yongzhou, PR China; e College of Food Science and Technology, Huazhong Agriculture University, Wuhan 430070, PR China; f Department of Environmental Agricultural Science, Faculty of Graduate Studies and Environmental Research, Ain Shams University, Cairo, Egypt; g Department of Food Science and Nutrition, College of Biosystems Engineering and Food Science, Zhejiang University, Hangzhou 310058, PR China

**Keywords:** Tea polysaccharides, Myofibrillar proteins, Surimi, Interaction profiling, Frozen storage

## Abstract

This study aimed to evaluate the protective effect of tea polysaccharides (TPR) on surimi quality during freeze–thaw cycles (FTC). Although TPR has shown cryoprotective potential, its effects on water mobility, thermal transitions, and protein interaction profiles in surimi under repeated FTC conditions remain unclear. Surimi and myofibrillar proteins were treated with TPR at 1% and 3%, an industrial mixture (CP), or no treatment (C) during FTC. In C samples, the gel strength decreased from 3043.38 to 1078.82 g·mm, with shifts in T21 and T22 relaxation behavior and Tg. Compared with TPR (1%), CP exhibited greater stability in Tg, T21/T22 relaxation behavior, and ΔH. Notably, TPR (3%) maintained water-state stability, gel strength, thermal stability, and an operational protein interaction profile under the FTC conditions. These findings suggest that 3% TPR may serve as a promising natural cryoprotectant for improving surimi quality during FTC.

## Highlights

• Water transitional changes were analyzed during freeze thaw cycles.
• Tea polysaccharides (TPR) (3%) maintained the water binding and gel strength.
• TPR (3%) improved the ionic and hydrogen contents among protein functional sites.
• TPR (3%) reduced the loosely bound water molecules in the surimi protein matrix.
• TPR mitigated the water molecules availability, resulting in microstructural damage.
• Overall, TPR (3%) maintained the surimi protein network against water transitions.

## 1 Introduction

*Hypophthalmichthys molitrix* is an important freshwater aquaculture species, accounting for a large proportion of fish production in Asia, with approximately 3.2 million metric tons produced annually in China (Jiang et al., 2025). The popularity of *H. molitrix* worldwide may stem from its rapid adaptability and growth, making it an important source of protein (Jiang et al., 2022). However, the postharvest processing of *H. molitrix* remains a significant challenge for the industry. Surimi, a concentrated myofibrillar protein (MPF) source, is a significant constituent of various surimi analogs owing to its key role in the structural and functional abilities of the final product (Maghsoudi et al., 2023). Maintaining the structural and functional attributes of surimi proteins is crucial during freeze–thaw cycles (FTC) and extended frozen storage. Freezing surimi is a widely adopted technique used at the industrial level during storage to reduce microbial and enzymatic activity and slow protein deterioration, including aggregation, oxidation, and denaturation. Meanwhile, cold storage and the FTC, including those that may occur during transportation and handling, can affect the structural and functional characteristics of proteins due to instability, leading to excessive formation of free water molecules and eventually, the development of ice crystals (Yang et al., 2025). According to earlier studies, even minor temperature fluctuations, such as ±2 °C, can promote ice crystal growth and damage the protein network. This change in the protein network can lead to the removal of free water molecules, creating an inferior gel network and harder texture, which are the key parameters of surimi products (Li et al., 2022; Yang et al., 2025).

Temperature variations are difficult to avoid during the commercialization and distribution of surimi products. For example, repeated changes in temperature during transportation from warehouses to retailers and markets, frequent opening of freezers, and malfunctioning of storage systems may cause partial thawing of surimi and related products. These frequent FTC can accelerate the conversion of smaller ice crystals to larger ice crystals, intensifying structural instability, as reported previously (Zhang et al., 2020). To prevent such changes in surimi proteins, industries are more inclined to use cryoprotectants, such as sucrose, phosphate and sorbitol. Indeed, these cryoprotectants have partial protective abilities against FTC, such as improving water-binding ability and metal chelation; however, their addition can result in metallic taste and health concerns regarding sweetness and sugar levels (Kuepethkaew et al., 2025). Therefore, these concerns have spurred interest in alternative natural cryoprotective additives for meat preservation. Recently, many studies have reported the utilization of different polysaccharides, oligosaccharides, peptides, and hydrolysates as potential cryoprotectants because of their hydrophilic groups and water-interaction capacity. However, the limited availability and high cost of some natural sources restrict their industrial applications (Yang et al., 2025). Tea polysaccharides (TPR), extracted from *Camellia sinensis*, are potential candidates because tea is widely available, and TPR contains abundant hydrophilic groups that may interact with water molecules and proteins through hydrogen-bonding interactions, thereby contributing to reduced water mobility and limited ice-crystal growth. As a continuation of our previous studies, the interaction mechanism between surimi proteins and TPR was further clarified in our earlier reports (Walayat et al., 2024; Walayat et al., 2025). The presence of hydrophilic moieties in TPR can stabilize the surimi protein network during FTC-induced temperature changes. It is also possible that, according to water replacement theory, TPR may help reduce dehydration-driven protein destabilization through possible potential interactions with water and protein functional groups. In addition, based on the glass transition hypothesis, TPR may increase matrix viscosity and reduce free water mobility, thereby potentially limiting ice crystal growth.

Although our previous studies reported the effects of tea polysaccharides on protein structural changes and intermolecular interactions in silver carp surimi under FTC, the relationship between water mobility, thermal transitions, and microstructural disruption associated with ice crystal damage has not yet been clearly established. Therefore, the novelty of the present study lies in the integration of differential scanning calorimetry, low-field nuclear magnetic resonance, operational protein interaction profiling, and light microscopy to clarify the mechanism by which TPR protects surimi quality during FTC. In addition, this study directly compared 1% and 3% TPR with an industrial cryoprotectant mixture under the same storage design, thereby identifying the more effective treatment for preserving water state stability and protein network integrity. We hypothesized that TPR, particularly at 3%, would reduce FTC-induced water mobility changes in surimi proteins, thereby preserving gel functionality, thermal stability, and the operational protein interaction profile compared with untreated samples, while CP served as an industrial reference treatment. Accordingly, LF-NMR-derived water mobility, particularly T21 and T22 relaxation behavior, was defined as the primary outcome, whereas gel strength, WHC, DSC thermal properties, operational protein interaction profiling, and light microscopy were used as supporting outcomes. Therefore, in this study, we investigated the protein changes, water mobility, and microstructural disruption associated with ice crystal damage through differential scanning calorimetry, low-field nuclear magnetic resonance, and light microscopy analyses of *H. molitrix* treated with TPR (1 and 3%) and an industrial mixture of sucrose and sorbitol during FTC. This study aimed to advance sustainable strategies for maintaining surimi protein quality under repeated FTC.

## 2 Materials and methods

### 2.1 Raw materials and reagents

Six silver carp (*Hypophthalmichthys molitrix*), averaging 28 cm in length and 0.8 kg in weight, were sourced from a fish market in Hangzhou, Zhejiang, China, and pooled to prepare a composite batch of surimi and myofibrillar proteins (MPF) for subsequent treatment allocation. Tea polysaccharides (20230910), with a molecular weight of 1020 MW, were procured from Shanghai Qianwei Co., Ltd. Food-grade monosaccharide (sorbitol) and disaccharide (sucrose) were obtained from Henan GP Chemicals Co. Ltd. General laboratory chemicals, including salts and buffers, were sourced from Sinopharm Chemical Co., Ltd.

### 2.2 Surimi and myofibrillar protein preparation

After procurement, the deceased fish were brought to the Laboratory of Tea Processing and Tea Comprehensive Utilization on ice blocks within 15–20 min to reduce any postmortem alterations. The fish processing involved cutting off the tails and heads and removing the scales. The fillets were prepared, and a mechanical mincer was used to mince them. Surimi was produced following the standardized protocol described in our previous publication (Walayat et al., 2024), with minor changes to ensure consistency. Myofibrillar proteins (MPF) were obtained using a dual buffer strategy, as reported by Benjakul and Bauer (2001). First, the surimi was homogenized and centrifuged (10,000 ×*g*, for 10 min, at 4 °C) with a 0.05 M (NaCl) low buffer to solubilize the sarcoplasmic proteins. The sample was then homogenized and centrifuged (10,000 ×*g* for 10 min at 4 °C) with a 0.45 M (NaCl) high buffer to isolate the MPF. These processes were repeated thrice to purify the MPF. Protein content was quantified using the biuret method proposed by Gornal et al. (1949), and the protein content in fresh MPF was 91 mg/mL.

### 2.3 Preparation of surimi and MPF samples

Preparation of surimi and MPF samples After surimi and MPF preparation, all samples were divided into four categories based on treatments: (1) surimi and MPF samples incorporated with an industrial mixture of 4% sucrose and 4% sorbitol as a positive control (CP); (2) low-dose incorporation of TPR (1%); (3) high-dose incorporation of 3% TPR; and (4) surimi samples without any treatment as the control (C). After treatment, the C, CP, TPR (1%), and TPR (3%) samples were placed in polythene bags and properly sealed, with three independent sealed packets prepared for each treatment as experimental replicates for each treatment. The MPF samples (C, CP, TPR (1%), and TPR (3%)) were tightly capped in 50 mL tubes and prepared as three independent experimental replicates per treatment. Each sealed tube was considered an experimental unit, and all analytical measurements were performed in triplicate as technical replicates.

### 2.4 Simulated freeze thaw cycle model

To examine the effect of repeated temperature changes on surimi quality, all groups were subjected to repeated FTC. The samples were stored at −18 °C for two days and then thawed at 4 °C for one day; this was defined as one cycle. The process was repeated for two, four, and six cycles, with three independent experimental replicates per group analyzed at each interval. Prior to analyses, surimi and MPF samples (C, CP, TPR (1%), and TPR (3%)) were equilibrated for 12 h at 4 °C to ensure uniform temperature distribution.

**Note:** This temperature-abuse design was used to simulate repeated freezing and thawing that may occur during transport, handling, and temporary storage interruptions, which can promote protein changes, water mobility alterations, and ice recrystallization in surimi.

### 2.5 Gel strength

Surimi gel was prepared following the procedure adopted by Jia et al. (2019), with some modifications. The surimi samples were mixed with NaCl (2.5%). Then, the surimi was stuffed into polyvinylidene (3 cm) and heated in a hot water-bath at 40 °C for 60 min, followed by 90 °C for half an hour. The gel strength was recorded using a texture analyzer with a P/0.25 probe at pre- and post-test speeds of 1 and 10 mm/s, and the trigger force was 5 g. The gel strength of each independent experimental replicate was measured in triplicate as technical replicates, and the mean value was recorded in g·mm for statistical analysis.

### 2.6 Water holding capacity (WHC)

The WHC of the surimi gel samples (C, CP, TPR (1%), and TPR (3%)) was analyzed according to the method described by Sun et al. (2024), with some modifications. Briefly, surimi (10 *g*) was weighed and centrifuged at 8000 ×*g* for 10 min at 4 °C to remove unbound water. The WHC (%) was determined using the following equation:

$$\mathrm{WHC}=\left(\text{Post}-\text{centrifuge Weight}/\mathrm{Pre}-\text{centrifuge weight}\right)\times 100$$

The WHC was measured in triplicate for each independent experimental replicate, and the mean value was used for statistical analysis.

### 2.7 Low field nuclear magnetic resonance (LFMR)

Water mobility in MPF samples (C, CP, TPR (1%), and TPR (3%)) was evaluated following the protocol proposed by Huang et al. (2025) using an LF-NMR analyzer (Niumag Electric Company, Shanghai, China). Samples in glass tubes (H:30 mm and d:11) underwent T_22_ relaxation measure transverse relaxation time (T2) measurements using the Carr–Purcell–Meiboom–Gill (CPMG) pulse sequence under standardized settings: 23.2 MHz proton resonance, 0.5 T magnetic field, 1 ms echo time, 5000 ms sampling interval, echo number 10,000, and pre-amplifier gain was 1. The data from four scans per sample were averaged to obtain one value for each experimental unit. The T2 relaxation curves were processed using multi-exponential inversion analysis to obtain the relaxation time distributions. The relaxation components were defined as T2b (0–10 ms), T21 (10–100 ms), and T22 (100–1000 ms), corresponding to tightly bound, immobilized, and free or weakly retained water, respectively. The corresponding peak area fractions were calculated as P2b, P21, and P22, and the signal fraction above 1000 ms was considered as highly mobile/free water.

### 2.8 Measurement of glass transition (Tg) and ΔH

The Tg and ΔH values of the fish meat paste were determined following the method described by Zhang et al. (2023), with some modifications. Approximately 10 mg of each sample was weighed into special DSC aluminum pans, hermetically sealed, and then loaded onto the DSC instrument at room temperature, using an empty pan as a reference. Nitrogen (50 mL/min) was used as the carrier gas. The DSC measurement procedure was as follows: the samples were cooled at 5 °C /min from ambient temperature to −90 °C, held for 20 min, and heated up to 40 °C from −90 °C. The samples were then cooled from room temperature to −90 °C at 5 °C/min, heated again to 40 °C at 5 °C/min, and held at 40 °C for 30 min. Finally, the samples were cooled to −90 °C at 5 °C /min and scanned at 5 °C /min to 50 °C for the final heating scan used for Tg and ΔH analysis. Tg was determined from the glass-transition region of the final heating thermogram, and ΔH was calculated by integrating the endothermic peak area and normalizing it to sample mass; therefore, ΔH was expressed as J/g. The melting-transition parameters, including Tonset, Tpeak/Tm, and Tendset, were obtained from the same final heating scan. All samples were analyzed in triplicate as technical replicates, and the mean values for each sample were used for analysis.

### 2.9 Protein interaction profiling

Changes in MPF interaction profiling were determined using the methodology described by Su et al. (2025). MPF samples (1 g) were taken and homogenized (1 min, 4 °C, and at 5000 rpm) to fully dissolve in 9 mL of the following buffers (0.05 M NaCl (A) → 0.6 M NaCl (B) → 0.6 M NaCl +1.5 M urea (C) → 0.6 M NaCl +8 M urea(D)). This sequential extraction assay was used to estimate the protein solubility fractions associated with different non-covalent interaction environments, rather than directly quantifying the molecular bond number or bond strength. After homogenization and centrifugation (10,000 ×*g* for 15 min at 4 °C), soluble protein levels were measured in the supernatants using the Lowry method. The operationally defined interaction-associated fractions were calculated as follows and expressed as mg soluble protein/mL:

• **Ionic interactions** = (Solution B – Solution A);
• **Hydrogen bonds** = (Solution C – Solution B);
• **Hydrophobic forces** = (Solution D – Solution C)

Because these values were derived from differences in soluble protein extracted under sequential solvent conditions, they were interpreted as indirect operational indicators of protein network changes. Each assay was performed in triplicate as technical replicates, and the mean value was used for the analysis.

### 2.10 Thermal transition

Protein The denaturation of proteins in MPF samples was analyzed using DSC (TA Instruments-Waters LLC, Model 250, New Castle, Delaware, USA) following the method described by Wu et al. (2020). At this stage, 10 mg of the sample was tightly sealed in an aluminum crucible pan. The parameters were set as follows: heating range and rate, 25–110 °C and 5 °C, respectively. An empty crucible pan was used as a blank. At least three samples were analyzed in parallel as technical replicates, and mean values were used for analysis.

### 2.11 Muscle fiber structural imaging

Light microscopy analysis was performed to analyze fish muscle disruption according to the method described by Jiang et al. (2024). Muscle fiber alignment and ice crystal morphology were visualized using a Leica CM 1850 cryostat (Leica, Wetzlar, Germany). Frozen surimi samples (0.5 × 0.5 × 1 cm3) were taken with transverse and longitudinal 4-μm sections, which were fixed, stained with eosin (15 min) and then HCl (1%) for ensuring uniform color over the samples. The samples were dehydrated in ethanol gradients (70, 80, 90, and 100%) and mounted in resin. Images captured under an Olympus microscope (Tokyo, Japan) were used to highlight the structural changes at 20× magnification induced by FTC. The micrographs shown in the revised figure were exported from the scanner fields and presented without additional color enhancement during figure assembly. For quantitative image analysis, three independent experimental replicates were examined per treatment and time point. ImageJ software was used to quantify the apparent microstructural disruption. The pseudo-colored micrographs were segmented using a fixed color-threshold method, in which the blue and green/cyan regions were defined as apparent void/disrupted regions. The area fraction, apparent ice crystal/void area, pore/void region count, mean and maximum apparent pore/void area, and mean and maximum equivalent pore diameter were calculated using the Analyze Particles function.

### 2.12 Statistical analysis

Statistical analysis was performed using the mean values obtained from three independent experimental replicates per treatment and FTC cycle. Technical triplicate readings were used only to calculate the mean for each experimental replicate and were not treated as independent observations. One-way ANOVA followed by Duncan's multiple range test was performed at *P* < 0.05 for two sets of simple-effect comparisons: (i) differences among treatments within the same FTC cycle and (ii) differences among FTC cycles within the same treatment. Therefore, the statistical conclusions were interpreted as simple effect comparisons rather than treatment × FTC interaction effects. Origin 2025 was used to generate the figures.

## 3 Results and discussion

### 3.1 Gel strength

Gel strength is an important indicator of the structural stability and functional quality of surimi. The impact of FTC (0 to 6) on the gel strength of C, CP, and TPR (1 and 3%) samples is shown in Table 1. FTC significantly reduced the gel strength of C, CP, and TPR (1 and 3%) despite their treatment with industrial and natural cryoprotectants. The C samples showed a marked decline in gel strength from 3043 to 1078 g·mm after 6 FTC. This reduced gel strength could be linked with FTC-induced protein destabilization, which may cause weaker gel formation and water loss. In addition, for the CP and TPR (1 and 3%) samples, a major drop was also noted in gel strength during these FTC. The CP and TPR (1%) were examined with a decline in gel strength (from 3051 to 1654 g·mm) and (from 3047 to 1463), respectively; however, this decline was more controlled than in the C samples. Interestingly, the CP samples showed a more restricted decline in gel strength than TPR (1%), which could be due to the cryoprotective effect of the sucrose-sorbitol mixture and its contribution to water retention and gel network development during heating. Furthermore, the inferior gel formation observed in TPR (1%) may be attributed to insufficient TPR concentration, weaker water-retention capacity, and less effective stabilization of the protein matrix. Moreover, after 6 FTC cycles, TPR (3%) showed higher gel strength retention than C, CP, and TPR (1%), declining from 3041 to 2029 g·mm (Table 1). This stability in gel strength may be associated with enhanced water retention and possible hydrogen bonding interactions between TPR, water molecules, and protein functional groups. These results are consistent with our findings, showing that TPR (3%) significantly maintained WHC (Fig. 1), water mobility (Fig. 2), interaction profiling (Fig. 4), and light microscopy analysis (Fig. 6), suggesting that TPR (3%) better limited water migration and FTC-induced protein-network disruption (Yang et al., 2025). In earlier studies, it was reported that the incorporation of naturally derived additives such as konjac oligo-glucomannan (Liu et al., 2019), carrageenan (Xia et al., 2023), and xylo-oligosaccharides (Zhang et al., 2020) in surimi or MPF can inhibit the decline in gel strength by enhancing water retention, improving cryoprotectant-protein interactions, and reducing ice-crystal-related damage to proteins.

**Table 1:** Gel strength of *H. molitrix* surimi treated with C, CP, TPR (1%) and TPR (3%) during FTC.

Gel Strength (g·mm)				
FTC/Treatments	C	CP	TPR (1%)	TPR (3%)
0	3043.12 ± 38.12^Aa^	3051.64 ± 56.24^Aa^	3047.51 ± 63.47^Aa^	3041.32 ± 68.23^Aa^
2	2634.52 ± 54.88^Ba^	2701.36 ± 47.35^Bb^	2742.63 ± 42.53^Bb^	2822.11 ± 62.43^Bc^
4	1716.29 ± 55.32^Ca^	2072.54 ± 61.09^Cb^	1926.58 ± 51.64^Cc^	2562.52 ± 49.46^Cd^
6	1078.82 ± 41.71^Da^	1654.45 ± 58.19^Db^	1463.85 ± 65.73^Dc^	2029.28 ± 64.89^Dd^

Letters (A–D) represent the individual treatment at different variations. Letters (a–d) represent the significance difference (*P* < 0.05) in different treatments within the individual variation. Error bars denote the standard deviation (SD) of the experimental units.

**Fig. 1:**
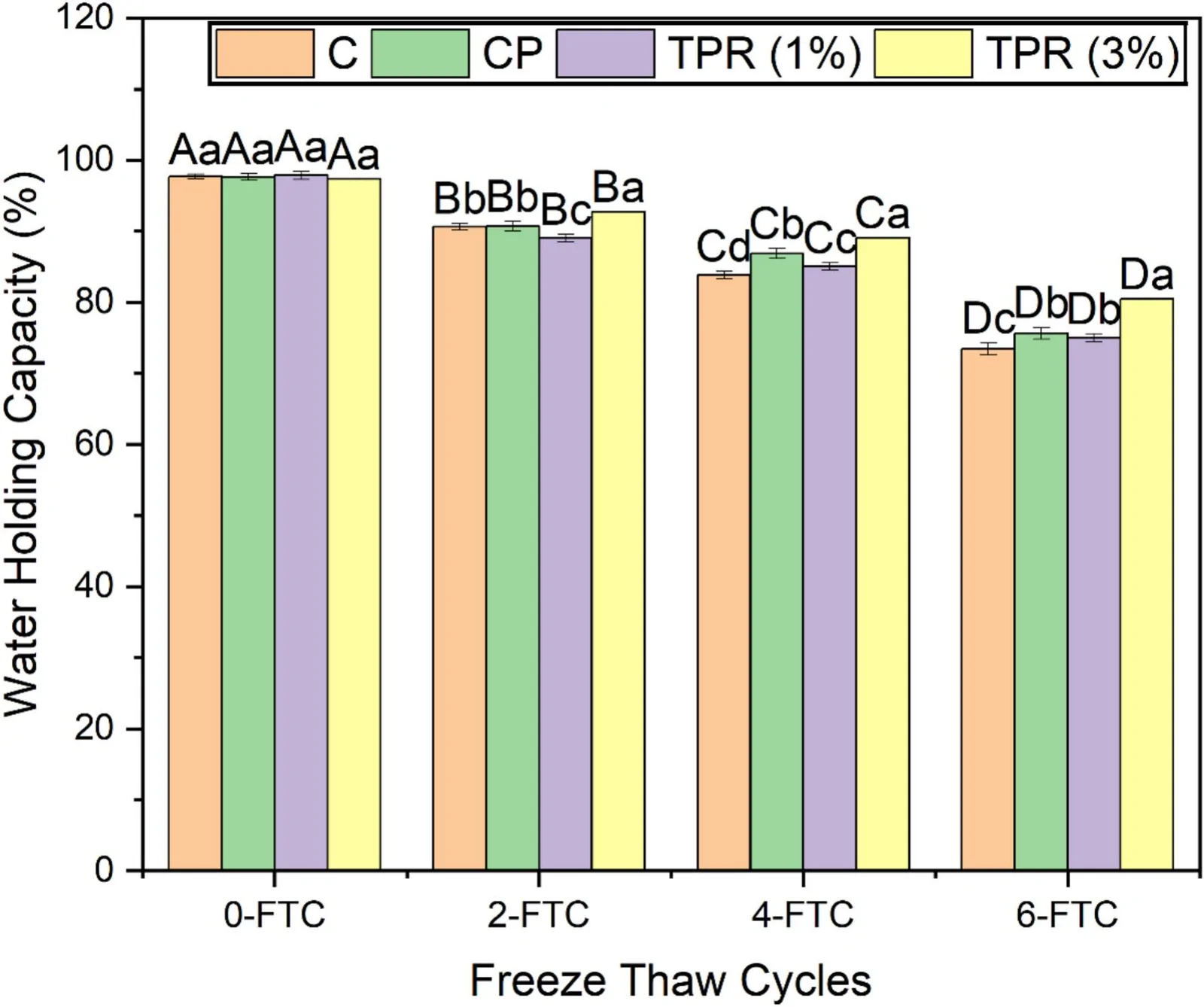
Water holding capacity of *H. molitrix* surimi gel treated with C, CP, TPR (1%) and TPR (3%) during FTC. Letters (A–D) indicate significant differences (*P* < 0.05) among different frozen storage variations within the same treatment, whereas letters (a–d) indicate significant differences (*P* < 0.05) among treatments within the same frozen storage variation. Error bars denote the standard deviation (SD) of the experimental units.

**Fig. 2:**
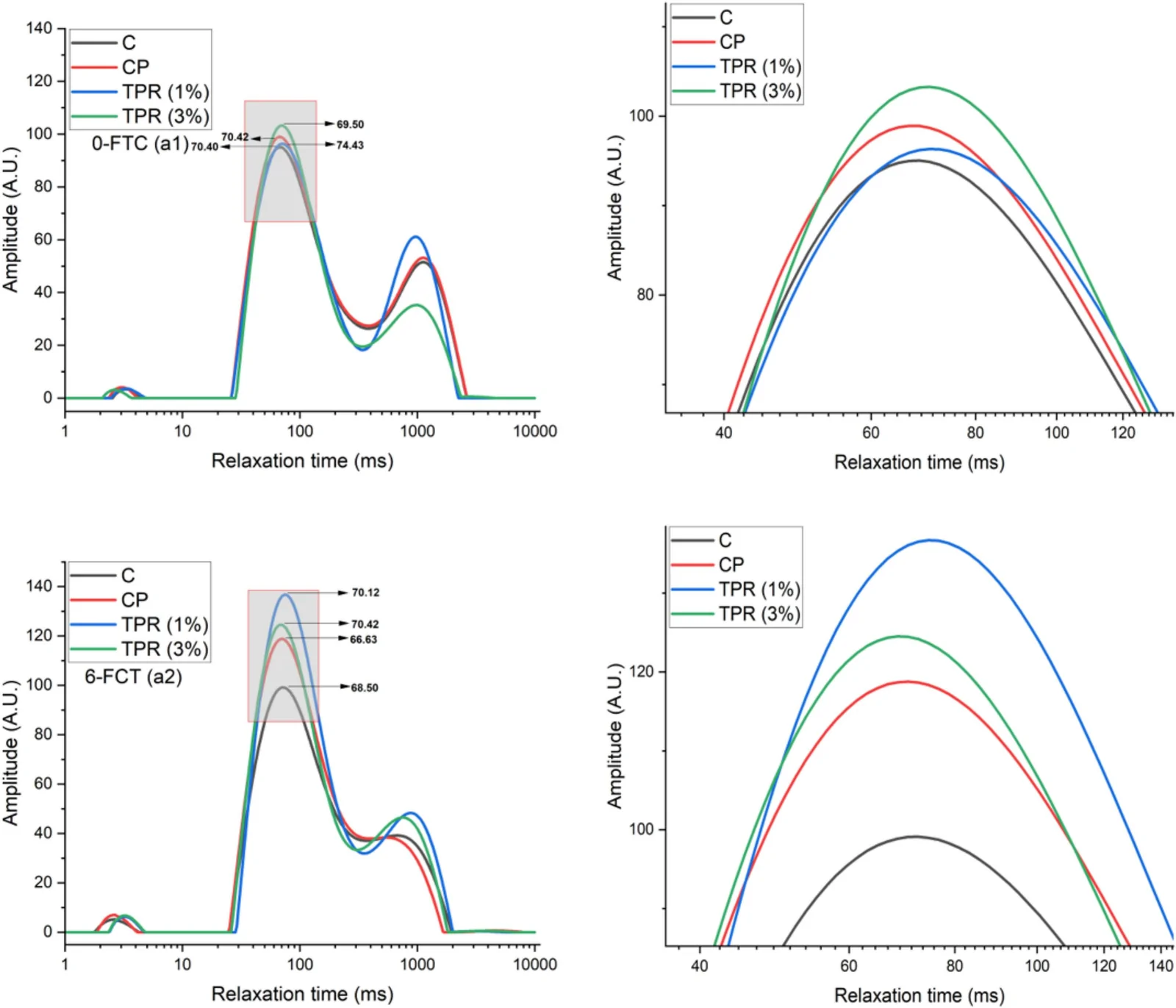
Water mobility of *H. molitrix* myofibrillar proteins treated with C, CP, TPR (1%) and TPR (3%) during FTC.

### 3.2 Water holding capacity (WHC)

WHC is an important indicator used to assess the moisture holding ability of surimi, and it is closely related to water migration, protein network integrity, and structural stability. The WHC of the surimi samples (C, CP, TPR (1%), and TPR (3%)) is shown in Fig. 1. The WHC decreased progressively during repeated FTC in all samples, although the extent of reduction differed among treatments. Initially, no significant differences were observed at 0 FTC, indicating that the freeze-thaw process had not yet caused measurable structural changes in the protein network. An actual decline in WHC was observed at 2 FTC in all samples (C, CP, TPR (1%), and TPR (3%)), as shown in Fig. 1. The samples with no treatments (C) demonstrated a major drop in WHC from 97.68 to 73.45% compared to the CP and TPR (1 and 3%) samples. Yang et al. (2025) stated that the decline in WHC could occur due to protein denaturation caused by the formation of larger ice crystals during frozen storage conditions, leading to disintegration in the protein structure and weakening functional sites. FTC can also promote water redistribution and reduce the ability of the protein matrix to retain water (Zhang et al., 2020). In addition, CP and TPR (1% and 3%) showed better WHC retention during FTC due to their interaction with protein sites. As shown in Fig. 1, the CP and TPR (1%) were analyzed along with a gradual decline in WHC, but the decline was less severe than that in the C samples. Notably, TPR (1%) showed a controlled decline in WHC from 97.88 to 74.93%, whereas CP declined from 97.63 to 75.64%. Thus, TPR (1%) and CP showed comparable WHC retention under the repeated FTC in this study. In addition to these CP and TPR (1%) treatments, the TPR (3%) concentration also resulted in a reduction in WHC from 97.32 to 80.43%; however, this decline was more gradual and better controlled than in the other treatments. These findings imply that TPR (3%) is more effective for maintaining water retention. Earlier research has established that the addition of natural cryoprotectants may maintain the WHC in surimi proteins by reducing FTC-induced water migration through possible interactions with water molecules (Sun et al., 2024). In another study, carrageenan addition prevented WHC decline by decreasing alterations in protein structure and securing myosin changes in MPF (Xia et al., 2023). Zhu et al. (2022) also reported that the incorporation of natural additives as cryoprotectants in surimi can prolong the structural integrity of proteins via possible hydrogen and ionic interactions with protein-associated groups, which may contribute to improved protein-matrix stability. These studies ensure the validity of our findings, suggesting that TPR at 3% can better restrict WHC loss by helping preserve the surimi protein network during FTC.

### 3.3 Water mobility in protein matrix

Water mobility within the protein system is important because of its association with the structural integrity, functionality, and overall quality of surimi. LF-NMR is a useful nondestructive technique for evaluating the distribution and mobility of water molecules within the protein matrices. The water mobilities of different samples, C, CP, and TPR (1 and 3%), are shown in Fig. 2. Based on the transverse relaxation time distribution, the LF-NMR signals were categorized into T2b (0–10 ms), T21 (10–100 ms), and T22 (100–1000 ms), corresponding to tightly bound water associated with hydrophilic sites, immobilized water retained within the protein network, and free or weakly retained water, respectively (Huang et al., 2025). Signals above 1000 ms were considered highly mobile/free water and are presented in Table S2. After 6 FTC cycles, the T2b fraction remained relatively small in all samples, indicating that tightly bound water represented only a minor proportion of the total water population. The most significant changes were observed in the T21 and T22 regions, reflecting the redistribution of immobilized and mobile water during the FTC. As shown in Fig. 2a1 and a2, the T21 peak positions changed after the 6 FTC. The T21 peak shifted from 70.40 to 68.50 ms in C, from 70.42 to 66.63 ms in CP, from 74.43 to 70.42 ms in TPR (1%), and from 69.50 to 70.12 ms in TPR (3%) (Table S2). These results indicate that TPR (3%) exhibited stable T21 relaxation behavior, suggesting better preservation of immobilized water within the MPF network. Quantitative peak area analysis further supported this trend. The immobilized-water fraction (P21) was maintained in TPR (3%), changing from 47.87% at 0 FTC to 48.09% at 6 FTC. In contrast, P21 decreased to 42.14%, 42.32%, and 42.23% in the C, CP, and TPR (1%) groups, respectively, after 6 FTC. This indicates that TPR (3%) was more effective in maintaining protein-water interactions and preventing the loss of immobilized water during FTC. The T22 region also showed clear changes after the FTC. The T22 peak shifted from 663.20 to 1122.28 ms in C and from 305.47 to 1122.28 ms in CP, indicating increased water mobility and greater conversion of retained water into a more mobile state in CP than in C. TPR (1%) and TPR (3%) exhibited lower T22 peak positions after 6 FTC, suggesting a more controlled water migration behavior than that of C and CP. Moreover, the highly mobile water fraction above 1000 ms increased to 14.93% in C and 14.90% in CP after 6 FTC, whereas it remained lower in TPR (1%) and TPR (3%) at 13.06% and 9.74%, respectively. The low highly mobile water fraction in TPR (3%) further confirms its ability to restrict water migration and maintain water retention within the protein matrix. This change in water distribution may be due to ice crystal damage, protein denaturation, and expulsion of water molecules from the protein matrix, resulting in reduced WHC (Fig. 1). The improved water stability observed in TPR (3%) may be attributed to hydrogen-bonding interactions between tea polysaccharides and protein functional groups, which could help stabilize the myofibrillar protein network and reduce FTC-induced water redistribution. These findings are consistent with the WHC (Fig. 1), gel strength (Table 1), and light microscopy observations (Fig. 6). Huang et al. (2025) reported that polysaccharides-maintained water mobility in frozen shrimp proteins by inhibiting protein unfolding and stabilizing the protein network. The addition of tea polysaccharides has also been reported to restrict water mobility by protecting myosin from oxidation-related changes, particularly in tryptophan and tyrosine residues (Walayat et al., 2024). Therefore**,** TPR (3%) effectively restricted changes in T21 and T22 relaxation behavior, maintained the immobilized-water fraction, and reduced the formation of highly mobile water, thereby protecting the protein matrix from FTC-induced water loss and deterioration of gelling properties.

### 3.4 Tg and ΔH via DSC

During frozen storage or FTC, proteins undergo several changes, including denaturation, oxidation, and aggregation. DSC was used to evaluate low-temperature thermal transitions by determining the glass transition temperature (Tg), melting-transition parameters, and enthalpy change (ΔH). The glass transition (Tg) and ΔH values of all samples treated with CP, TPR (1 and 3%), and C without any treatment are shown in Fig. 3 (a1 and a2), and the calculated Tonset, Tpeak/Tm, Tendset, Tg, and ΔH values are summarized in Table S3. During 0 FTC, there was a slight difference in Tg among all samples, ranging from −19.91 to −22.58 °C, while ΔH ranged from 1.54 to 2.08 J/g. In addition, the untreated samples (C) demonstrated a significant change in Tg from −20.83 to −27.08 °C after 6 FTC. Meanwhile, ΔH decreased from 1.76 to 1.67 J/g in C samples after the completion of 6 FTC. This decrease in Tg, together with the reduction in ΔH, indicates altered water-transition behavior and weakened thermal stability caused by repeated FTC. In contrast, the CP-treated samples showed a decrease in Tg from −19.91 to −24.75 °C, whereas TPR (1%) and TPR (3%) showed Tg changes from −22.58 to −24.37 °C and from −20.22 to −22.41 °C, respectively. Notably, TPR (3%) maintained the least negative Tg after 6 FTC (−22.41 °C), suggesting better stabilization of the unfrozen matrix than C, CP, and TPR (1%). The melting-transition parameters also support this trend. After 6 FTC, Tonset, Tpeak/Tm, and Tendset were −3.67, 1.58, and 7.67 °C in C; −3.67, 1.58, and 7.67 °C in CP; −4.00, 0.08, and 2.92 °C in TPR (1%); and −3.92, 0.92, and 3.92 °C in TPR (3%), respectively. Changes in ΔH were also observed for the CP and TPR treatments. For CP, ΔH decreased from 1.54 to 1.33J/g, whereas TPR (1%) decreased from 1.69 to 1.25J/g. Notably, TPR (3%) was effective in maintaining ΔH, which decreased only from 2.08 to 1.89 J/g. The higher ΔH retention in the TPR group (3%) suggests that this treatment maintained the water-associated thermal transition and reduced FTC-induced disruption of the protein-water matrix. The improved Tg and ΔH stability in TPR (3%) may be related to hydrogen bonding and ionic interactions between TPR, water molecules, and MPF functional groups, which may reduce unbound water mobility and limit ice-crystal growth. The controlled thermal attributes of TPR (3%) may also reflect better preservation of the structural and functional attributes of the protein matrix, as reported in our earlier study (Walayat et al., 2024). Moreover, previous research has reported that cryoprotectants can stabilize Tg and ΔH by reducing water mobility (Chen et al., 2013). These findings are consistent with the LF-NMR results (Fig. 2), WHC results (Fig. 1), and light microscopy observations (Fig. 6) of this study. Therefore, the corrected DSC results indicate that TPR (3%) maintained Tg and ΔH more effectively than C, CP and TPR (1%) during FTC, supporting its role in reducing water transition, limiting FTC-induced damage, and preserving surimi protein stability.

**Fig. 3:**
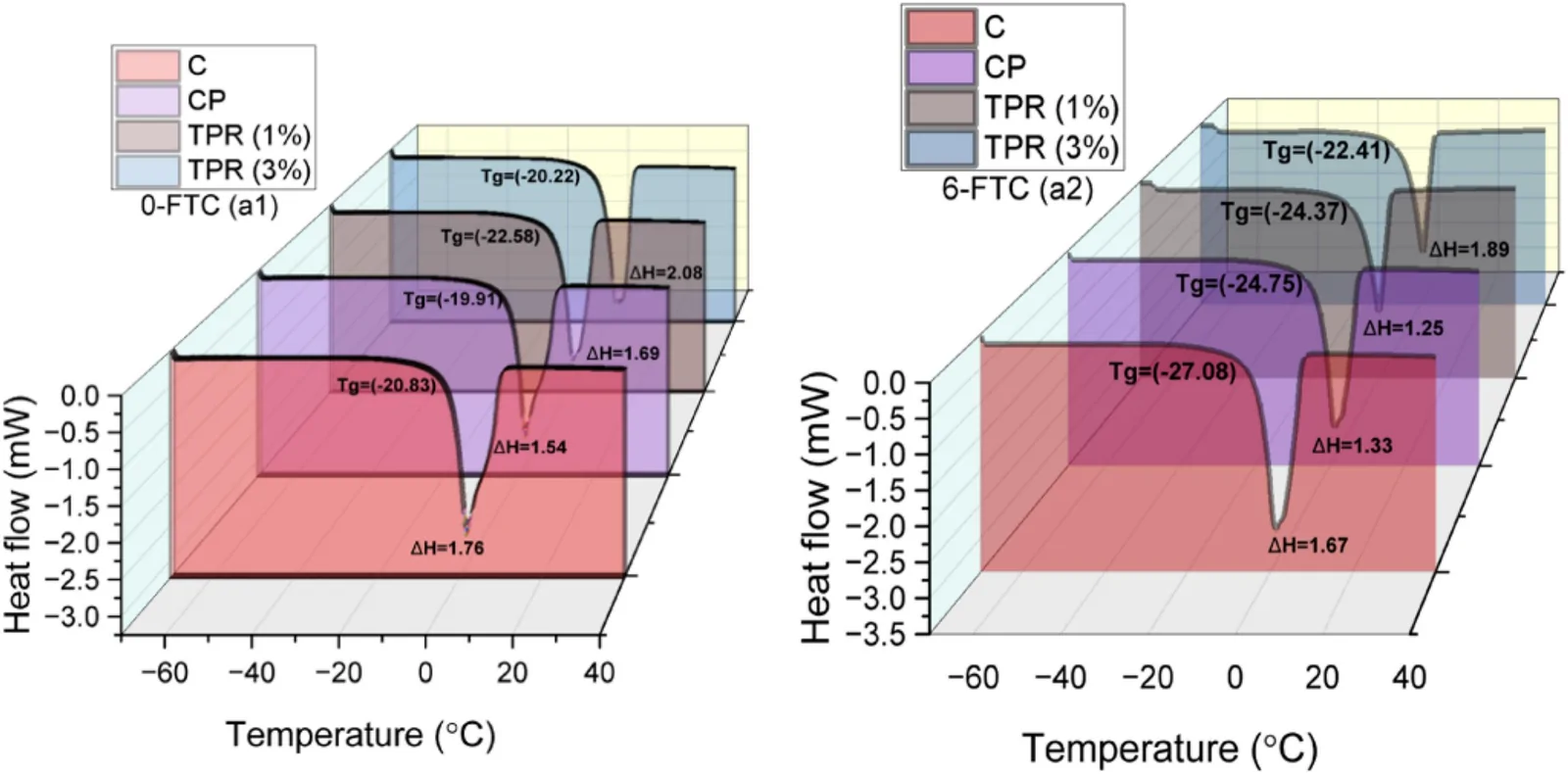
Tg and ΔH changes in *H. molitrix* myofibrillar proteins treated with C, CP, TPR (1%) and TPR (3%) during FTC.

### 3.5 Protein interaction profiling

Sequential extraction was used to evaluate operational changes in protein solubility associated with different non-covalent interaction environments. Because this assay is extraction-based, the resulting values should be interpreted as operational indicators of protein network changes rather than direct measurements of molecular bond strength. This operational protein interaction profile is relevant to gel formation and structural stability in surimi and related products. Protein interaction profiling of all samples (C, CP TPR (1 and 3%)) changed during the 6 FTC (Fig. 4a1, a2, and a3). All samples demonstrated an increase in hydrophobic-interaction-associated fraction, followed by a decline in ionic and hydrogen-associated fractions, despite being treated with CP and TPR (1 and 3%). During this operational protein interaction profile, C samples showed a substantial reduction in the ionic-interaction-associated fractions (from 1.02 to 0.28 mg soluble protein/mL) and hydrogen-bond-associated fractions (from 0.69 to 0.33 mg soluble protein/mL), as well as an increase in the hydrophobic-interaction-associated fractions (from 2.65 to 5.43 mg soluble protein/mL) after 6 FTC. Meanwhile, a decline was also noted for CP and TPR (1%) in the ionic- and hydrogen-interaction-associated fractions, but the decrease was sharper between these two treatments, from 1.04 to 0.45 mg soluble protein/mL and 1.07 to 0.42 mg soluble protein/mL, respectively. Despite this, CP showed better stability in the operational protein interaction profile than TPR (1%). Furthermore, these findings of ionic and hydrogen-associated fractions were consistent with an increase in the hydrophobic-interaction-associated fraction between CP (2.61 to 4.72 mg soluble protein/mL) and TPR (1%) increased from 2.59 to 4.83 mg soluble protein/mL after 6 FTC. Moreover, TPR (3%) also showed a decline in the interaction profiling ionic content (from 1.04 to 0.64 mg soluble protein/mL) and hydrogen content (from 0.67 to 0.43 mg soluble protein/mL), and an increase in the hydrophobic-interaction-associated fraction (from 2.67 to 4.21 mg soluble protein/mL). The changes in TPR (3%) were more restricted than those in C, CP, and TPR (1%), indicating a better retention of the operational protein interaction profile. These findings suggest that the addition of TPR as a natural additive could enhance the operational protein interaction profile of surimi proteins during repeated FTC and extended frozen storage. As a continuation of our previous study, in which the interaction mechanism of TPR with *H. molitrix* myofibrillar proteins was determined, the better retention of the operational protein interaction profile, including ionic, hydrogen bond, and hydrophobic interaction-associated fractions, suggests that TPR may help moderate electrostatic repulsion and protein network destabilization during FTC (Walayat et al., 2025). The better retention of the hydrogen-bond-associated fraction in TPR (3%) suggests improved protein network stability and may be associated with a stronger three-dimensional gel network (Chen et al., 2020). As a continuation of our previous study, these findings of structural stability in surimi proteins are also well evidenced in the earlier study by Walayat et al. (2024), in which the addition of TPR(3%) significantly maintained secondary structural attributes (α-helix, β-sheets, β-turn, and random coils) after 6 FTC. These interaction profiling outcomes also coincide with improved WHC (Fig. 1), gel strength (Table 1), and light microscopy imaging (Fig. 6). Moreover, the improved operational protein interaction profile could be due to TPR containing more hydroxyl groups, which may interact with available free water molecules via hydrogen bonding, thereby reducing protein-water interactions and water mobility within the protein matrix. Su et al. (2025) stated that cryoprotectants can uphold the interaction profile of surimi proteins by maintaining the sulfhydryl and hydrophobic groups and their exposure to polar environments, allowing amino acids to expand and establish a better gel network. The maintained operational protein interaction profile could be associated with the possible hydrogen-bonding interactions of TPR with water molecules, which may reduce water mobility within the protein matrix (Maghsoudi et al., 2023). Therefore, TPR (3%) better maintained the operational protein interaction profile, particularly the ionic- and hydrogen-interaction-associated fractions, suggesting improved protein-network stability and gel formation during FTC.

**Fig. 4:**
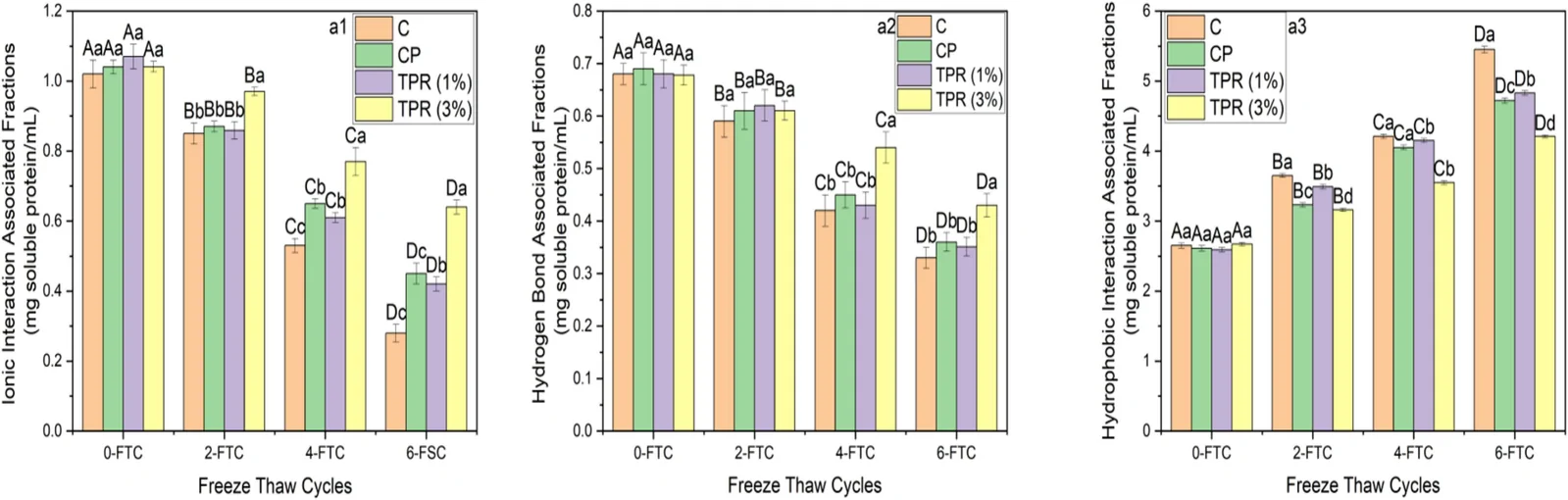
Operational protein interaction profiling of *H. molitrix* proteins treated with C, CP, TPR (1%) and TPR (3%) during FTC. The values represent operationally defined ionic-interaction-associated, hydrogen-bond-associated, and hydrophobic-interaction-associated protein fractions calculated from sequential extraction. Letters (A–D) represent significant differences among FTC cycles within the same treatment. Letters (a–d) represent significant differences (*P* < 0.05) among treatments within the same FTC. Error bars denote the standard deviation (SD) of the experimental units.

### 3.6 DSC thermal transition

Generally, the gelling attributes of surimi are based on the stability of MPF during processing. It can be established that the overall behavior and stability of MPF components are reduced in surimi due to the FTC. Moreover, due to changes in surimi storage conditions, the denaturation temperatures of different proteins may differ widely. To assess protein denaturation, thermal transition can be performed to analyze the peaks at which the ordered network unfolded and the breakdown of hydrogen bonds occurred. Usually, DSC thermal transition can be examined based on changes in transition at three different ranges, such as the myosin head (40–50 °C), myosin tail (55–60 °C), and actin at 70–80 °C, respectively (Ren et al., 2023). Based on previous results of WHC, LF-NMR, Tg, and light microscopy analysis, no significant changes were observed among C, CP, and TPR (1 and 3%) recorded at 0 FTC; therefore, thermal transition was performed at 6 FTC among all treatments to analyze the effect of thermal denaturation in all treated samples and the cryoprotective role in protein stability. The changes in the thermal transitions of C, CP, and TPR (1 and 3%) are presented in Fig. 5. A significant shift in the thermal transition peak in the C samples can be observed at the peak from 45 to 50 °C compared to CP and TPR (1 and 3%), indicating that the myosin head region had already denatured due to FTC (Wei et al., 2018). Meanwhile, a transition shift at 60 °C and 70 °C indicates the loss of myosin tail and actin, representing reduced gel formation, strength, and destabilization in C samples. In addition, the TPR (1%) sample was also analyzed with a change in transition at 45 to 50 °C, but it was not as prominent as in the C samples, suggesting partial protection of the myosin head region (Xiong et al., 2023). Moreover, for TPR (1%), changes in the peak were observed at 60 and 70 °C. Apart from these cases, the CP-and TPR (3%)-treated surimi samples showed similar thermal transition peaks at 40–50 °C, 60 °C, and 71.53 °C, suggesting better retention of thermal-transition behavior associated with the myosin and actin regions (Xiong et al., 2023). The more stable thermal transition behavior in CP, especially TPR (3%), indicates better preservation of the protein network, reduced crystal-induced destabilization, and improved stability of MPF during FTC. These results support the cryoprotective effect of TPR (3%), which may help preserve protein thermal stability during FTC.

**Fig. 5:**
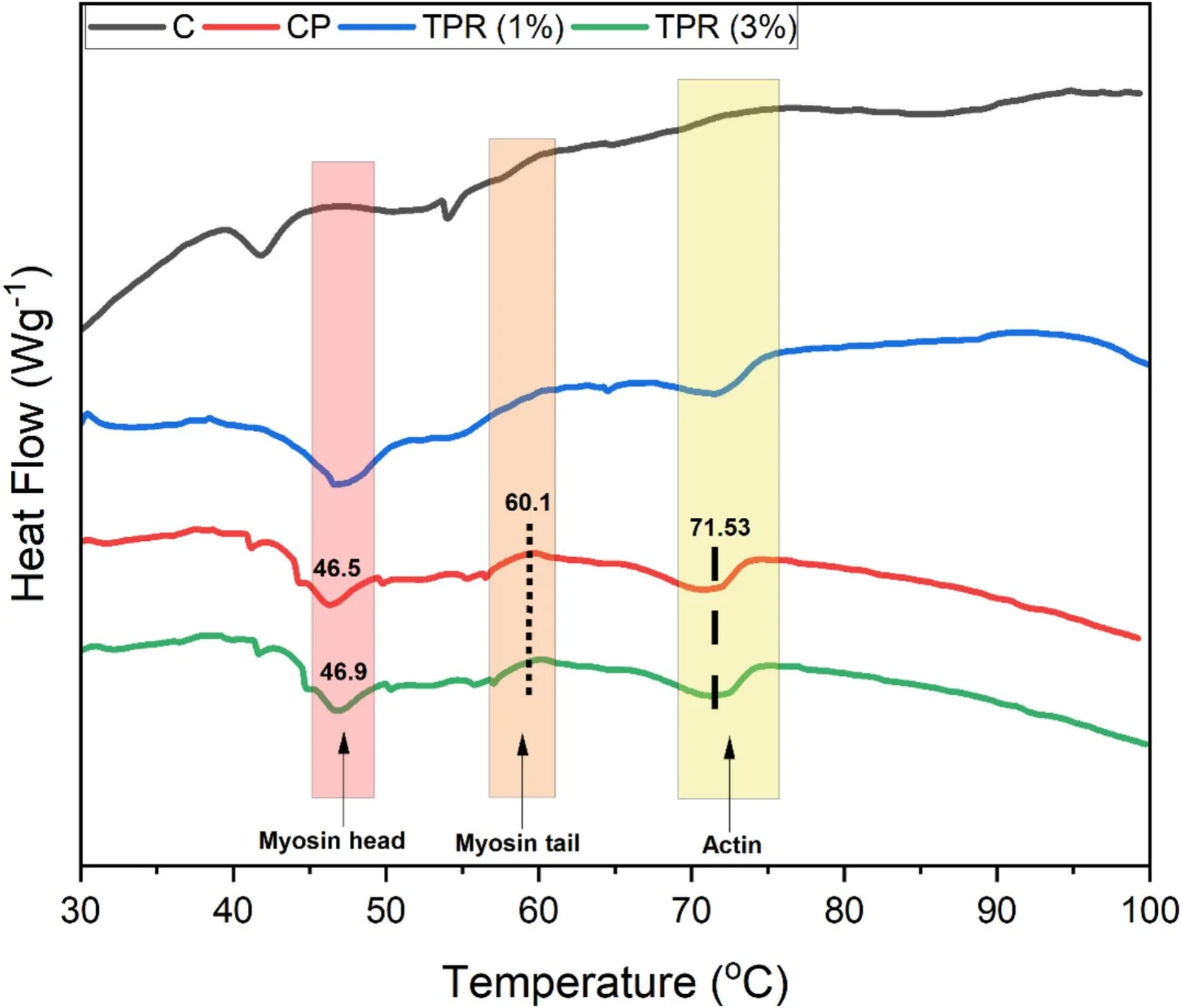
Thermal transitional changes in *H. molitrix* myofibrillar proteins treated with C, CP, TPR (1%) and TPR (3%) during FTC.

### 3.7 Light microscopy analysis

Light microscopy was used to evaluate the microstructural disruption associated with FTC-induced ice crystal damage. The changes in the muscles are shown in Fig. 6a1 and a2. To support the visual observations, the relative disrupted/void area (%) was quantified from the displayed fields. At 0 FTC, all samples remained compact, whereas after 6 FTC, the disrupted/void area increased markedly in C (9.57%) and TPR (1%) (9.76%), but remained lower in CP (5.91%) and TPR (3%) (4.62%), indicating better preservation of the protein matrix by the latter treatments (Table S1). Additional image analysis indices further supported this pattern. The apparent ice crystal/void area increased from 1384.0 to 9832.2 μm^2^ in C and from 717.2 to 10,026.4 μm^2^ in TPR (1%) after 6-FTC, whereas lower values were observed in CP (6067.3 μm^2^) and TPR (3%) (4745.4 μm^2^). Similarly, the maximum apparent pore/void area was highest in TPR (1%) (2858.9 μm^2^), followed by TPR (3%) (1007.3 μm^2^), C (852.2 μm^2^), and CP (700.3 μm^2^), indicating that TPR (3%) reduced the overall void fraction, although some localized disrupted regions remained. The mean equivalent pore diameter after 6 FTC was also lower in TPR (3%) (2.56 μm) and CP (2.65 μm) than in C (3.13 μm) and TPR (1%) (3.87 μm), confirming that CP and TPR (3%) better limited pore enlargement and microstructural disruption. A certain level of damage was observed in all samples (C, CP, TPR (1 and 3%)) after 6 FTC cycles, as shown in Fig. 6a1. During 0 FTC, no significant changes were noted in C, CP, and TPR (1 and 3%) treated samples. The lack of alterations at 0 FTC in all samples could be linked with no FTC initiation and insufficient time for the additives to bind with surimi proteins based on their interaction processes, such as ionic, hydrogen, and hydrophobic interactions. The change in muscle protein disintegration in all samples (C, CP, TPR (1 and 3%)) can be visualized after 6 FTC (Fig. 6a2). Major changes in protein matrix damage, network disruption, and denaturation were recorded in the C sample during light microscopy analysis, visualizing the impact of crystallization damage after 6 FTC. This marked change in the C samples indicates the formation of ice crystals and their conversion into larger ice crystals, which may have caused larger pores and void spaces in the protein matrix.

**Fig. 6:**
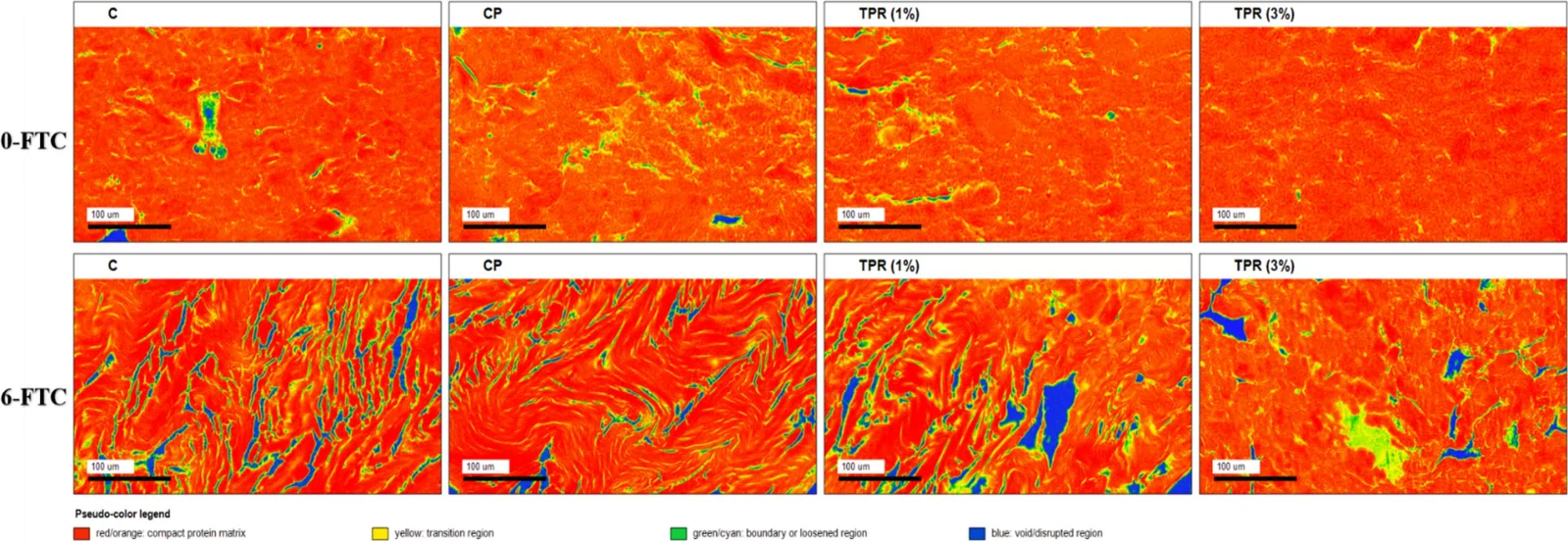
Light microscopic analysis of *H. molitrix* muscles treated with C, CP, TPR (1%) and TPR (3%) during FTC.

These changes in muscle fibers can be associated with the loss of ionic- and hydrogen-interaction-associated fractions and increased availability of loosely bound water, resulting in reduced gel strength (Table 1) and WHC (Fig. 1). Muscle fiber denaturation was also noted in CP and TPR (1 and 3%), but this denaturation was less pronounced than in the C samples, indicating that CP and TPR assisted in the preservation of the protein network and maintained the protein structure. Notably, CP provided better protection than TPR (1%) against FTC-induced structural changes, possibly due to stronger cryoprotective interactions and a more effective barrier against protein destabilization. Thus, TPR (1%) appeared to be a less effective concentration, although it still provided some protection during FTC. Meanwhile, TPR (3%) showed a more intact protein network in surimi after 6 FTC cycles (Fig. 6a1 and a2) than CP and TPR (1%), and its performance was better than that of CP for several structural indicators. This better-preserved microstructure may reflect the cryoprotective effect of 3% TPR, which likely reduced protein instability associated with the redistribution of loosely bound water and ice-crystal-related protein damage (Zhu et al., 2022). These quantified microstructural changes were consistent with the LF-NMR, Tg, gel strength, and operational protein interaction profile results, confirming that TPR (3%) reduced FTC-induced changes. Moreover, TPR may also contribute to protein-structure preservation, as reported in our earlier publications, and may be relevant to the molecular interaction mechanisms described in our previous docking and simulation study (Walayat et al., 2025). More importantly, these findings confirm our outcomes from WHC (Fig. 1), gel strength (Table 1), Tg (Fig. 3), and operational protein interaction profile (Fig. 4). Earlier studies have stated that crystallization and recrystallization in proteins could be linked with temperature fluctuations and the extracellular environment (Zhang et al., 2019). During Frozen storage, recrystallization may concentrate the extracellular solution, resulting in MPF dehydration and a decrease in the freezing temperature (Yang et al., 2025). In the current study, TPR (3%) appeared to reduce the microstructural disruption associated with crystallization/recrystallization during FTC, (evidenced by Tg (Fig. 3) and LF-NMR (Fig. 2)). Therefore, the present findings support the role of TPR (3%) in limiting protein-network disintegration associated with recrystallization and maintaining microstructural stability.

## 4 Conclusion

The primary outcome, LF-NMR-derived water mobility, supported the hypothesis that TPR (3%) reduced FTC water transition, while the supporting measurements confirmed the associated improvements in gel functionality, thermal stability, protein interactions, and microstructural integrity. This study analyzed the effects of TPR (1% and 3%) on water mobility, thermal transitions, and structural stability in *H. molitrix* surimi during 6 FTC cycles, compared with an industrial mixture (CP) and untreated samples (C). The results showed that FTC-induced marked changes in the C samples, as evidenced by Tg, LF-NMR, reduced gel strength, and lower WHC, which were associated with altered protein dynamics, increased unbound water, and weaker protein interactions. In contrast, CP and TPR treatments better preserved thermal stability and reduced FTC-induced damage to surimi muscle fibers than the other treatments. Notably, CP exhibited stronger cryoprotective effects than TPR (1%), whereas TPR (3%) showed improved protective effects compared to CP for several measured endpoints, including water mobility, thermal stability, gel strength, and microstructural preservation. Compared with our previous publications, this study extends the available knowledge by linking water mobility, thermal transitions, and microstructural changes under FTC, thereby providing additional evidence for the cryoprotective action of the TPR. Overall, TPR (3%) may be a promising natural cryoprotectant for surimi under the FTC conditions; however, its industrial suitability cannot yet be confirmed without sensory, color, flavor, microbial, shelf-life, regulatory, cost, and process-scale validation. Future studies should address these practical aspects before firm industrial conclusions are drawn. The present study is also limited by its laboratory-scale design, use of a single fish species and fixed storage protocol, and absence of sensory, microbiological, and pilot-scale processing data; therefore, the findings should be interpreted as condition-specific rather than universally generalizable.

## CRediT authorship contribution statement

**Noman Walayat:** Writing – original draft, Formal analysis. **Piotr Kulawik:** Writing – review & editing. **Isam A. Mohamed Ahmed:** Writing – review & editing, Resources. **Asad Nawaz:** Software, Data curation. **Nadia Niaz:** Conceptualization. **Mahmoud Salah:** Writing – review & editing. **Muhammad Naveed Sheas:** Methodology. **Zhucheng Su:** Supervision. **Ran Wei:** Funding acquisition.

## Funding statement

This research was funded by Ongoing Research Funding program (ORF-2026-1074), King Saud University, Riyadh, Saudi Arabia.

## Declaration of competing interest

The authors declare that they have no known competing financial interests or personal relationships that could have appeared to influence the work reported in this paper.

## Data Availability

Data will be made available on request.
