# Supplementary material for: Tea polysaccharides improved the water mobility, thermal stability and microstructural changes of Hypophthalmichthys molitrix surimi during frozen thaw cycles
Source: Food Chem X. 2026 Sep 22;39:104498. doi: 10.1016/j.fochx.2026.104498 (PMC13634727; doi:10.1016/j.fochx.2026.104498)
Supplement: Supplementary file 1 [file mmc1.docx]

**Table S1. Quantitative image data of light microscopic analysis**

| **Treatment** | **FTC** | **Relative disrupted/void area (%)** | **Apparent ice-crystal/void area (um²)** | **Void/pore region count** | **Mean apparent pore/void area (um²)** | **Maximum apparent pore/void area (um²)** | **Mean equivalent pore diameter (um)** | **Maximum equivalent pore diameter (um)** |
| --- | --- | --- | --- | --- | --- | --- | --- | --- |
| C | 0-FTC | 1.35 | 1384.0 | 82 | 16.88 | 373.0 | 2.80 | 21.79 |
| CP | 0-FTC | 1.23 | 1260.3 | 105 | 12.00 | 270.4 | 2.95 | 18.55 |
| TPR (1%) | 0-FTC | 0.70 | 717.2 | 63 | 11.38 | 118.8 | 3.02 | 12.30 |
| TPR (3%) | 0-FTC | 0.04 | 44.8 | 8 | 5.60 | 18.5 | 2.42 | 4.86 |
| C | 6-FTC | 9.57 | 9832.2 | 464 | 21.19 | 852.2 | 3.13 | 32.94 |
| CP | 6-FTC | 5.91 | 6067.3 | 445 | 13.63 | 700.3 | 2.65 | 29.86 |
| TPR (1%) | 6-FTC | 9.76 | 10026.4 | 279 | 35.94 | 2858.9 | 3.87 | 60.33 |
| TPR (3%) | 6-FTC | 4.62 | 4745.4 | 300 | 15.82 | 1007.3 | 2.56 | 35.81 |

**Table S2.** Quantitative LF-NMR relaxation parameters and peak-area fractions of surimi/MPF samples treated with C, CP, TPR (1%), and TPR (3%) during freeze thaw cycles.

| **Cycle** | **Treatment** | **T2b peak (ms)** | **T21 peak (ms)** | **T22 peak (ms)** | **P2b (%)** | **P21 (%)** | **P22 (%)** | **Mobile water >1000 ms (%)** |
| --- | --- | --- | --- | --- | --- | --- | --- | --- |
| 0-FTC | C | 2.61 | 70.40 | 663.20 | 1.24 | 44.00 | 48.70 | 6.06 |
| 0-FTC | CP | 2.68 | 70.42 | 305.47 | 1.40 | 48.26 | 46.88 | 3.46 |
| 0-FTC | TPR (1%) | 3.26 | 74.43 | 874.75 | 1.02 | 45.33 | 46.25 | 7.40 |
| 0-FTC | TPR (3%) | 3.26 | 69.50 | 740.87 | 1.21 | 47.87 | 45.18 | 5.74 |
| 6-FTC | C | 3.26 | 68.50 | 1122.28 | 0.56 | 42.14 | 42.38 | 14.93 |
| 6-FTC | CP | 3.08 | 66.63 | 1122.28 | 0.66 | 42.32 | 42.11 | 14.90 |
| 6-FTC | TPR (1%) | 3.35 | 70.42 | 977.19 | 0.68 | 42.23 | 44.02 | 13.06 |
| 6-FTC | TPR (3%) | 2.68 | 70.12 | 977.19 | 0.56 | 48.09 | 41.62 | 9.74 |

T2b, T21, and T22 represent tightly bound water, immobilized water, and free/weakly retained water, respectively. P2b, P21, and P22 indicate the corresponding peak-area fractions. The fraction above 1000 ms represents highly mobile/free water.

**Table S3.** DSC-derived glass transition temperature, melting-transition parameters, and enthalpy change of H. molitrix surimi samples treated with C, CP, TPR (1%), and TPR (3%) during freeze thaw cycles

| **Cycle** | **Treatment** | **Tg (°C)** | **Tonset (°C)** | **Tpeak/Tm (°C)** | **Tendset (°C)** | **ΔH (J/g)** |
| --- | --- | --- | --- | --- | --- | --- |
| 0-FTC | C | −20.83 | −3.00 | 1.67 | 9.25 | 1.76 |
| 0-FTC | CP | −19.91 | −3.00 | 1.67 | 9.25 | 1.54 |
| 0-FTC | TPR (1%) | −22.58 | −3.67 | 1.58 | 7.67 | 1.69 |
| 0-FTC | TPR (3%) | −20.22 | −3.50 | 1.92 | 7.75 | 2.08 |
| 6-FTC | C | −27.08 | −3.67 | 1.58 | 7.67 | 1.67 |
| 6-FTC | CP | −24.75 | −3.67 | 1.58 | 7.67 | 1.33 |
| 6-FTC | TPR (1%) | −24.37 | −4.00 | 0.08 | 2.92 | 1.25 |
| 6-FTC | TPR (3%) | −22.41 | −3.92 | 0.92 | 3.92 | 1.89 |

Tg = glass transition temperature; Tonset = onset temperature of the melting transition; Tpeak/Tm = peak melting temperature; Tendset = endset temperature of the melting transition; ΔH = enthalpy change calculated from the integrated endothermic peak area and expressed as J/g.
